# BAFF polymorphisms and serum levels of BAFF in Tunisian systemic lupus erythematosus patients

**DOI:** 10.1186/1479-5876-10-S3-P35

**Published:** 2012-11-28

**Authors:** Nacira Laamiri, Imen Sfar, Tarak Dhaouadi, Lamia Ben Hassine, Salwa Jendoubi-Ayed, Awatef Chiha, Thouraya Ben Romdhane, Mouna Makhlouf, Taïeb Ben Abdallah, Narjess Khalfallah, Khaled Ayed, Yousr Gorgi

**Affiliations:** 1 Research Laboratory of Renal Transplantation and Immunopathology (LR03SP01), University Tunis El Manar, Charles Nicolle Hospital, Tunisia; 2 Dept. of Medicine, Charles Nicolle Hospital, Tunis, Tunisia

## Introduction

Although different authors suggest that the B-lymphocyte activating factor (BAFF) may be involved in the selective loss of B-cell tolerance in human systemic lupus erythematosus (SLE), the mechanisms responsible for the deregulation of this molecule in SLE remain unclear [1-3].

## Aims

To investigate any associations between regulatory genetic polymorphisms of BAFF gene, disease susceptibility and serum BAFF (s-BAFF) levels in Tunisian systemic lupus (SLE) patients.

## Methods

The case-control study included 124 SLE patients and 152 healthy controls. Three single nucleotide polymorphisms (SNPs) (-2841 T>C, -2701 A>T and -871 C>T) in the 5’ regulatory region of the BAFF gene were explored by PCR-RFLP [4]. s-BAFF levels were measured by ELISA (R&D Systems).

## Results

s-BAFF levels were elevated in SLE patients (1717,08 pg/ml) and in anti-dsDNA positive antibodies patients (1948,28 pg/ml) compared to both controls (665,82 pg/ml, p<10^-3^) and patients without anti-dsDNA antibodies (1281,51 pg/ml, p:0,007). In contrast, no correlation was found between global disease activity registered in SLEDAI and s-BAFF levels (p: 0.7). Furthermore, no association was found between BAFF genotypes and susceptibility to SLE. Single allele, genotype and haplotype association analyses showed no significant association with s-BAFF values, clinical features or SLEDAI score in SLE.

## Conclusions

Polymorphisms in the regulatory region of the BAFF gene do contribute neither to increased s-BAFF levels nor to the susceptibility to SLE in Tunisian patients. Increased s-BAFF levels in anti-dsDNA positive antibodies SLE patients suggest the central role of this molecule in the inflammatory process involving in autoantibodies production.
